# Analysis of the sex-specific variability of blood parameters in data sets of the Mouse Phenome Database

**DOI:** 10.1186/s13104-021-05739-w

**Published:** 2021-08-21

**Authors:** Bernhard Aigner

**Affiliations:** Chair for Molecular Animal Breeding and Biotechnology, and Laboratory for Functional Genome Analysis (LAFUGA), Gene Center, LMU Munich, 81377 Munich, Germany

**Keywords:** Animal model, Clinical chemistry, Hematology, Sex, Variability

## Abstract

**Objective:**

The use of mice as animal models in biomedical research allows the standardization of genetic background and environmental conditions, which both affect phenotypic variability. As the use of both sexes in experiments is strongly recommended, sex-specific phenotypic variability is discussed with regard to putative consequences on the group size which is necessary for achieving valid and reproducible results. In this study, the sex-specific variability of 25 clinical chemical and hematological parameters which represent a comprehensive blood screen of laboratory mice, was analyzed in data sets which have been submitted to the Mouse Phenome Database.

**Results:**

The overall analysis comprising all 25 clinical chemical and hematological parameters showed no evidence for substantial and robust general sex-specific variability. A large range of the ratio of the female and male coefficient of variation (CV) was found for every parameter among the respective strain data sets. This clearly demonstrated the appearance of unpredictable major interactions between genotype and environment regarding the sex-specific variability of the blood parameters analyzed.

## Introduction

When carrying out biomedical research with animal models, the extent of the phenotypic variability affects the group size which has to be used in the experiment for achieving valid and reproducible results. The use of both sexes in the experiments is strongly recommended because of possible differences in the outcome [1, 2]. Therefore, the appearance of sex-specific phenotypic variability is discussed in the context of differences in the social status especially in male group housed mice as well as of non-synchronous hormone cycles in female experimental groups which may alter the homogeneity of study populations and subsequently confound effects of experimental manipulations ([3–6] and refs. therein).

Meta-analyses examined published mouse data concerning this topic [4, 5, 7]. Prendergast et al. examined behavioral, morphological, physiological, and molecular traits in 293 articles, which were monitored in male mice and females tested without regard to the estrous cycle stage. The variability was not significantly greater in females than males for any endpoint and was substantially greater in males for several traits. In addition, group housing of mice was observed to increase the variability in both males and females by 37% [4].

In this study, the sex-specific variability of 25 clinical chemical and hematological parameters of laboratory mouse strains was analyzed in data sets which have been submitted to the Mouse Phenome Database. The blood parameters represent a comprehensive blood screen and were chosen as quantitative parameters for the analysis because they reflect a large range of phenotype traits.

## Main text

### Methods

The following ontology terms (VT, vertebrate trait ontologies) were used for the selection of the project data sets of the chosen parameters in the Mouse Phenome Database (https://phenome.jax.org): cholesterol, VT:0000180; creatinine, VT:0005328; glucose, VT:0000188; total protein, VT:0005567; triglycerides, VT:0002644; urea, VT:0005265; uric acid, VT:0010302; ferritin, VT:0010513; transferrin, VT:0010514; calcium, VT:0001562; chloride, VT:0003018; phosphorus, VT:0001565; potassium, VT:0002668; sodium, VT:0001776; alanine aminotransferase (ALT, EC 2.6.1.2), VT:0001573; aspartate aminotransferase (AST, EC 2.6.1.1), VT:0000203; α-amylase (EC 3.2.1.1), VT:0010475; alkaline phosphatase (AP, EC 3.1.3.1), VT:0000202; creatine kinase (CK, EC 2.7.3.2), VT:1000047; lipase (EC 3.1.1.3), VT:0010478; hemoglobin, VT:0001588; mean corpuscular volume (MCV), no VT applied; red blood cell count (RBC), VT:0001586; white blood cell count (WBC), VT:0000217; platelets, VT:0003179. The parameters hematocrit, mean corpuscular hemoglobin (MCH), and mean corpuscular hemoglobin concentration (MCHC) were not included in the study as they are subsequently calculated by using parameters which were directly measured.

Within each selected project data set, strain data sets were chosen for the analysis of the coefficient of variation (CV = standard deviation/mean) of males and females within the same data set by using following selection criteria: inbred strains [including those derived from the Collaborative Cross (CC)], F1 hybrids, recombinant inbred strains; no lines with newly generated alleles; no treatment; age of the mice examined: 7–26 weeks; group size: n ≥ 5 for each sex; as of December 2020.

Strain data sets with unusually high phenotypic variability for a given parameter due to e.g. technical outliers or unrecognized reasons—which was defined by the appearance of CV values > 0.5 for the female and/or the male group—were excluded from the further analysis. This mainly occurs for the three parameters creatinine, ALT and CK. For all other parameters, 2.3% (1.1% female group outliers, 0.6% male group outliers, and 0.6% outliers in both the female and male groups) of the strain data sets were excluded as outliers. The additional analysis with all strain data sets included did not change the results described below. Data analysis was carried out using the software program Microsoft Excel 2016 (Microsoft Corp., Redmond, WA). The chi-squared test was used for the statistical analysis of the data.

### Results

The phenotypic variability of 25 clinical chemical and hematological parameters was analyzed by determining the coefficient of variation (CV = standard deviation/mean) both for the male and female mice within a given strain data set. The extent of the CV value for both sexes depends on the phenotypic parameter analyzed (e.g. the red blood cell parameters hemoglobin, MCV and RBC as well as the electrolytes calcium, chloride and sodium usually exhibit relatively low CV values; and the enzyme activities ALT, AST and CK as well as the plasma substrate triglycerides exhibit relatively high CV values). Within a given strain data set, a CV ratio (= female CV/(female CV + male CV)) < 0.5 indicates that the female CV is lower than the male CV, whereas a CV ratio > 0.5 indicates that the female CV is higher than the male CV.

The data analysis was first carried out by using all strain data sets where at least five mice were examined for each sex. For the 25 chosen blood parameters, the mean CV ratios ranged from 0.44 to 0.53. Twelve parameters showed a mean CV ratio > 0.5, whereas the other twelve parameters showed a mean CV ratio < 0.5 (no data sets were included for the parameter CK by the selection procedure described in “Methods” section). For a given parameter, the portion of strain data sets with a CV ratio > 0.5 ranged from 24 to 75%. The parameter MCV showed an inconsistency of the mean CV ratio vs. the portion of strain data sets showing a CV ratio > 0.5 or < 0.5, respectively (i.e. a mean CV ratio of 0.49, whereas 55% of the strain data sets showed a CV ratio > 0.5) (Table 1).

**Table 1:** Coefficient of variation ratios [= female CV/(female CV + male CV)] of blood parameter data sets submitted to the Mouse Phenome Database (https://phenome.jax.org)

Parameter	n ≥ 5/sex						n ≥ 10/sex					
	Mean CV ratio ± SD	CV ratio > 0.5: % (n)	CV ratio < 0.5: % (n)	Range of CV ratio (min–max)	n mice (f + m)	n of project data sets	Mean CV ratio ± SD	CV ratio > 0.5: % (n)	CV ratio < 0.5: % (n)	Range of CV ratio (min–max)	n mice (f + m)	n of project data sets
Cholesterol	**0.52** ± 0.11	66% (220)	34% (111)	0.13–0.82	6461	17	**0.51** ± 0.11	58% (62)	42% (44)	0.21–0.79	3198	12
Creatinine	0.48 ± 0.10	50% (6)	50% (6)	0.23–0.65	482	4	0.498 ± 0.07	44% (4)	56% (5)	0.40–0.65	424	3
Glucose	**0.51** ± 0.10	56% (142)	44% (110)	0.21–0.93	4657	17	**0.51** ± 0.07	58% (31)	42% (22)	0.36–0.69	1949	9
Total protein	0.49 ± 0.08	41% (48)	59% (70)	0.24–0.73	2760	9	0.496 ± 0.08	49% (23)	51% (24)	0.31–0.72	1555	6
Triglycerides	**0.51** ± 0.11	56% (163)	44% (126)	0.15–0.86	5654	16	**0.51** ± 0.10	56% (57)	44% (44)	0.25–0.74	2922	10
Urea	**0.52** ± 0.09	64% (112)	36% (64)	0.19–0.82	3726	12	**0.51** ± 0.10	59% (29)	41% (20)	0.23–0.73	1750	8
Uric acid	0.49 ± 0.11	45% (21)	55% (26)	0.29–0.73	989	3	0.48 ± 0.09	40% (6)	60% (9)	0.35–0.63	403	3
Ferritin	**0.53** ± 0.07	75% (3)	25% (1)	0.43–0.58	119	1	**0.53** ± 0.07	75% (3)	25% (1)	0.43–0.58	119	1
Transferrin	0.46 ± 0.06	25% (1)	75% (3)	0.38–0.51	119	1	0.46 ± 0.06	25% (1)	75% (3)	0.38–0.51	119	1
Calcium	0.497 ± 0.11	48% (80)	52% (86)	0.22–0.84	3644	12	0.49 ± 0.11	45% (33)	55% (40)	0.28–0.78	2101	8
*Chloride*	***0.51*** ± 0.10	57% (66)	43% (50)	0.20–0.86	2346	7	*0.46* ± 0.16	40% (6)	60% (9)	0.20–0.86	783	4
*Phosphorus*	***0.51*** ± 0.08	54% (62)	46% (53)	0.21–0.72	2651	9	*0.49* ± 0.08	46% (21)	54% (25)	0.32–0.71	1474	6
Potassium	**0.51** ± 0.07	60% (70)	40% (47)	0.17–0.68	2307	7	**0.51** ± 0.07	55% (6)	45% (5)	0.42–0.64	682	4
*Sodium*	***0.51*** ± 0.10	62% (73)	38% (44)	0.20–0.79	2308	7	*0.48* ± 0.14	33% (4)	67% (8)	0.21–0.79	712	4
ALT	0.44 ± 0.11	24% (5)	76% (16)	0.25–0.70	416	5	0.39 ± 0.11	10% (1)	90% (9)	0.25–0.56	265	3
AST	0.49 ± 0.08	47% (30)	53% (34)	0.20–0.71	1319	6	0.49 ± 0.08	50% (10)	50% (10)	0.32–0.60	540	4
α-Amylase	**0.51** ± 0.09	55% (32)	45% (26)	0.28–0.67	1251	3	**0.51** ± 0.11	46% (11)	54% (13)	0.28–0.67	610	3
*AP*	*0.496* ± 0.10	46% (43)	54% (50)	0.24–0.78	1955	7	***0.51*** ± 0.08	59% (16)	41% (11)	0.37–0.76	825	4
CK	–						–					
Lipase	0.49 ± 0.13	41% (9)	59% (13)	0.30–0.76	347	1	–					
Hemoglobin	0.48 ± 0.12	47% (159)	53% (177)	0.07–0.88	6774	16	0.48 ± 0.11	46% (57)	54% (68)	0.14–0.88	3721	11
MCV	0.49 ± 0.12	55% (183)	45% (149)	0.09–0.90	7075	16	0.496 ± 0.12	57% (69)	43% (52)	0.09–0.80	4031	11
RBC	0.49 ± 0.11	49% (154)	51% (163)	0.08–0.85	6354	15	0.47 ± 0.10	36% (40)	64% (71)	0.27–0.78	3396	10
WBC	**0.52** ± 0.10	60% (210)	40% (142)	0.18–0.86	7050	17	**0.501** ± 0.08	56% (69)	44% (55)	0.28–0.72	3690	11
*Platelets*	***0.51*** ± 0.11	55% (183)	45% (150)	0.17–0.89	6732	16	*0.49* ± 0.10	52% (64)	48% (60)	0.26–0.76	3703	11

CV: coefficient of variation = standard deviation/mean; CV ratio: coefficient of variation ratio [= female CV/(female CV + male CV)]; SD: standard deviation. The values of the mean CV ratios are indicated in bold for the parameters where the female CV is higher than the male CV (i.e. a CV ratio > 0.5)

“n ≥ 5/sex” and “n ≥ 10/sex”: data of “n ≥ 10/sex” is included in the data of “n ≥ 5/sex”. For each parameter, the number of strain data sets included in the analysis and showing a CV ratio > 0.5 and a CV ratio < 0.5 is given in parentheses behind the respective value in %. Exclusion of strain data sets was carried out where the female and/or the male group showed a CV > 0.5 for the respective parameter

n of project data sets: the number of project data sets from which strain data sets were included in the analysis

Parameters and their results are depicted in smaller characters in the case that less than 50 strain data sets were available for the analysis “n ≥ 5/sex”. This refers to the six parameters creatinine, uric acid, ferritin, transferrin, ALT and lipase

The parameters chloride, phosphorus, sodium, AP and platelets and their mean CV ratios are indicated in italics as they showed inconsistent CV ratios in both analyses, i.e. a CV ratio > 0.5 in the analysis “n ≥ 5/sex” and a CV ratio < 0.5 in the analysis “n ≥ 10/sex” appeared; or vice versa

*ALT* alanine aminotransferase (EC 2.6.1.2), *AST* aspartate aminotransferase (EC 2.6.1.1); α-amylase (EC 3.2.1.1), *AP* alkaline phosphatase (EC 3.1.3.1), *CK* creatine kinase (EC 2.7.3.2); lipase (EC 3.1.1.3), *MCV* mean corpuscular volume, *RBC* red blood cell count, *WBC* white blood cell count

Subsequently, the data analysis was carried out by using only strain data sets where at least ten mice were examined for each sex. For the 25 chosen blood parameters, the mean CV ratios ranged from 0.39 to 0.53. Nine parameters showed a mean CV ratio > 0.5, whereas 14 parameters showed a mean CV ratio < 0.5. A relatively high deviation from the hypothesized CV ratio of 0.5 (i.e. a CV ratio < 0.48 or > 0.52) was found for the parameters ferritin (mean CV ratio = 0.53 of 4 data sets), transferrin (mean CV ratio = 0.46 of 4 data sets), chloride (mean CV ratio = 0.46 of 15 data sets), ALT (mean CV ratio = 0.39 of 10 data sets), and RBC (mean CV ratio = 0.47 of 111 data sets). Thus, except of the parameter RBC, in all other cases only a low number of strain data sets was available. For a given parameter, the portion of strain data sets with a CV ratio > 0.5 ranged from 10 to 75%. The parameters α-amylase, MCV and platelets showed an inconsistency of the mean CV ratio vs. the portion of strain data sets showing a CV ratio > 0.5 or < 0.5, respectively (Table 1).

The comparison of the outcome of the data analyses “n ≥ 5/sex” vs. “n ≥ 10/sex” revealed inconsistent CV ratios in both analyses, i.e. a CV ratio > 0.5 in the analysis “n ≥ 5/sex” and a CV ratio < 0.5 in the analysis “n ≥ 10/sex” or vice versa for the five parameters chloride, phosphorus, sodium, AP and platelets. Compared to the hypothesis of equal numbers of strain data sets with a CV ratio < 0.5 and a CV ratio > 0.5, the statistical examination of the detected counts of strain data sets with a CV ratio > 0.5 and with a CV ratio < 0.5 was carried out by using the chi-squared test to detect a consistent significant difference (p < 0.05) in both analyses “n ≥ 5/sex” and “n ≥ 10/sex” for a given parameter. This was only found for the parameter ALT; a parameter with only a low number of strain data sets available. In addition, large ranges (minimum–maximum) of the CV ratio appeared for more or less all parameters in both analyses “n ≥ 5/sex” and “n ≥ 10/sex” (Table 1).

Subsequently, the data sets were analyzed with respect to the particular inbred strains which were identified as the most often examined strains for the 25 blood parameters. Again, only data sets with at least five mice for each sex examined were included. Here, strain data sets were not excluded in the case that the female and/or the male group shows a CV > 0.5 for a given parameter. For each of the six inbred strains A/J, BALB/c, C3H, C57BL/6, CBA and DBA/2J, ten or more parameters were achieved with results from five or more different project data sets. The numbers of parameters showing a mean CV ratio > 0.5 were as follows for the particular inbred strains: 9 out of 10 for A/J, 4 out of 14 for BALB/c, 6 out of 12 for C3H, 11 out of 19 for C57BL/6, 6 out of 12 for CBA, and 6 out of 10 for DBA/2J (Table 2). Due to the low number of parameters with at least five data sets included in the analysis, the results may only provide hints for the potential appearance of sex-specific variability in particular blood parameters and/or specific strains.

**Table 2:** Coefficient of variation ratios [= female CV/(female CV + male CV)] of blood parameter data sets of selected inbred strains submitted to the Mouse Phenome Database (https://phenome.jax.org)

Mean CV ratio ± SD (n of data sets)	A/J…	BALB/c…	C3H…	C57BL/6…	CBA/…	DBA/2J…
Cholesterol	**0.56** ± 0.09 (9)	**0.54** ± 0.05 (13)	**0.53** ± 0.10 (10)	**0.51** ± 0.13 (16)	**0.51** ± 0.16 (8)	**0.61** ± 0.12 (7)
Creatinine	0.49 ± 0.02 (2)	**0.50** ± 0.06 (5)	0.48 ± 0.04 (3)	**0.51** ± 0.08 (6)	**0.53** ± 0.06 (3)	0.50 (1)
Glucose	**0.60** ± 0.10 (7)	0.45 ± 0.12 (11)	**0.52** ± 0.05 (8)	**0.52** ± 0.09 (17)	0.48 ± 0.06 (6)	**0.59** ± 0.10 (5)
Total protein	0.49 ± 0.04 (3)	0.50 ± 0.04 (9)	0.43 ± 0.11 (6)	**0.50** ± 0.07 (7)	0.47 ± 0.04 (5)	**0.55** ± 0.07 (4)
Triglycerides	**0.55** ± 0.10 (9)	0.50 ± 0.14 (13)	**0.54** ± 0.07 (10)	**0.52** ± 0.16 (16)	**0.55** ± 0.10 (8)	**0.54** ± 0.12 (7)
Urea	**0.50** ± 0.18 (5)	0.45 ± 0.11 (11)	**0.56** ± 0.09 (8)	**0.52** ± 0.12 (10)	**0.54** ± 0.10 (6)	**0.52** ± 0.15 (5)
Uric acid	0.44 (1)	0.48 ± 0.01 (4)	0.46 (1)	**0.52** ± 0.10 (3)	**0.55** ± 0.12 (3)	**0.67** (1)
Ferritin	–	**0.58** (1)	**0.53** (1)	**0.58** (1)	–	–
Transferrin	–	**0.51** (1)	0.50 (1)	0.46 (1)	–	–
Calcium	0.49 ± 0.08 (5)	0.47 ± 0.11 (11)	0.49 ± 0.11 (8)	**0.52** ± 0.08 (10)	0.47 ± 0.13 (6)	**0.56** ± 0.13 (6)
Chloride	**0.53** ± 0.06 (3)	**0.61** ± 0.17 (4)	**0.61** ± 0.18 (4)	**0.51** ± 0.13 (5)	**0.50** (1)	**0.53** ± 0.06 (2)
Phosphorus	**0.54** ± 0.05 (3)	0.46 ± 0.11 (9)	**0.52** ± 0.07 (6)	**0.53** ± 0.18 (7)	0.48 ± 0.08 (5)	0.48 ± 0.09 (4)
Potassium	**0.54** ± 0.06 (3)	**0.54** ± 0.04 (4)	**0.50** ± 0.07 (4)	0.46 ± 0.04 (5)	**0.53** (1)	0.47 ± 0.03 (2)
Sodium	0.48 ± 0.01 (3)	**0.55** ± 0.05 (4)	**0.64** ± 0.15 (4)	**0.52** ± 0.10 (5)	**0.51** (1)	0.46 ± 0.04 (2)
ALT	0.47 (1)	0.46 ± 0.08 (4)	0.41 ± 0.04 (3)	0.45 ± 0.09 (5)	0.40 ± 0.22 (2)	0.40 ± 0.11 (2)
AST	0.45 ± 0.21 (3)	**0.54** ± 0.04 (4)	0.46 ± 0.01 (2)	0.42 ± 0.09 (5)	**0.51** ± 0.01 (3)	0.47 (1)
α-Amylase	**0.55** ± 0.05 (2)	**0.54** ± 0.09 (4)	**0.53** ± 0.06 (2)	0.50 ± 0.10 (4)	**0.57** ± 0.02 (3)	0.34 (1)
AP	**0.50** ± 0.04 (3)	**0.56** ± 0.10 (5)	**0.57** ± 0.19 (3)	**0.53** ± 0.16 (5)	0.44 ± 0.00 (3)	**0.53** ± 0.07 (2)
CK	–	**0.52** ± 0.05 (4)	0.43 ± 0.10 (3)	**0.52** ± 0.14 (3)	0.42 ± 0.22 (2)	0.39 ± 0.08 (2)
Lipase	0.44 (1)	**0.58** (1)	0.40 (1)	–	–	0.43 (1)
Hemoglobin	**0.51** ± 0.17 (10)	0.47 ± 0.12 (15)	0.46 ± 0.07 (10)	0.45 ± 0.08 (15)	0.50 ± 0.07 (9)	0.45 ± 0.08 (8)
MCV	**0.56** ± 0.10 (10)	**0.51** ± 0.14 (15)	0.49 ± 0.14 (10)	0.46 ± 0.08 (15)	**0.51** ± 0.12 (11)	0.45 ± 0.13 (8)
RBC	**0.52** ± 0.12 (9)	0.45 ± 0.12 (14)	0.49 ± 0.06 (9)	0.48 ± 0.09 (14)	**0.52** ± 0.09 (10)	0.39 ± 0.11 (7)
WBC	**0.51** ± 0.11 (11)	0.48 ± 0.11 (16)	0.45 ± 0.08 (11)	0.50 ± 0.10 (16)	**0.53** ± 0.05 (11)	**0.50** ± 0.07 (9)
Platelets	**0.56** ± 0.14 (10)	0.47 ± 0.13 (15)	**0.51** ± 0.11 (10)	0.49 ± 0.11 (15)	0.49 ± 0.10 (10)	0.47 ± 0.07 (8)
“n ≥ 5”: n CV ratio > 0.5	9 of 10	4 of 14	6 of 12	11 of 19	6 of 12	6 of 10
“n ≥ 1”: n CV ratio > 0.5	14 of 22	13 of 25	12 of 25	14 of 24	13 of 22	10 of 23

CV: coefficient of variation = standard deviation/mean; CV ratio: coefficient of variation ratio [= female CV/(female CV + male CV)]; SD: standard deviation. The values of the mean CV ratios are indicated in bold for the parameters where the female CV is higher than the male CV (i.e. a CV ratio > 0.5). No exclusion of strain data sets was carried out in the case that the female and/or the male group showed a CV > 0.5 for the respective parameter

Data sets with the designation A/J…, BALB/c…, C3H…, C57BL/6…, CBA/… and DBA/2J… were summarized for the respective inbred strains A/J, BALB/c, C3H, C57BL/6, CBA and DBA/2 J

“n ≥ 5” and “n ≥ 1”: the number of data sets available for a given parameter of a selected inbred strain is shown in parentheses behind the value of the mean CV ratio ± SD. Results including less than five data sets are depicted in smaller characters. The analysis “n ≥ 5” exhibits the number of parameters with a CV ratio > 0.5 compared to the number of all parameters with at least five data sets. The analysis “n ≥ 1” exhibits the number of parameters with a CV ratio > 0.5 compared to the number of all parameters with at least one data set

*ALT* alanine aminotransferase (EC 2.6.1.2), *AST* aspartate aminotransferase (EC 2.6.1.1); α-amylase (EC 3.2.1.1), *AP* alkaline phosphatase (EC 3.1.3.1), *CK* creatine kinase (EC 2.7.3.2); lipase (EC 3.1.1.3), *MCV* mean corpuscular volume, *RBC* red blood cell count, *WBC* white blood cell count

### Discussion

The Mouse Phenome Database (https://phenome.jax.org) provides reference values of a high number of phenotype parameters mostly for the inbred strains which are predominantly used in biomedical research. Therefore, the data sets from this database were chosen for the analysis of sex-specific variability. The overall analysis comprising all 25 clinical chemical and hematological parameters showed no evidence for a substantial general sex-specific variability.

It is assumed that the data sets have been achieved by using the housing method which is usually carried out when working with mice in biomedical research, i.e. both sexes are group housed, and females are used without regard to the stage of the estrous cycle. The group sizes of the strain data sets selected for this study may fit to the relatively low numbers of animals which are usually used as group size at least in fundamental biomedical research. A meta-analysis revealed that group housing of mice increased the variability in both males and females by 37% [4]. Therefore, no sex is expected to take advantage of this housing method in respect to the extent of the variability compared to the other sex. The similar increase of the variability in group housed males and females is also expected to cover the consequences of the Lee Boot effect which leads to the suppression of the estrous cycle in group housed female mice [8].

The analysis of the sex-specific variability of 25 blood parameters resulted in a relatively high deviation from the hypothesized CV ratio of 0.5 (i.e. a CV ratio < 0.48 or > 0.52) only for the parameter RBC (mean CV ratio = 0.47 of 111 data sets in the analysis “n ≥ 10/sex”) where a sufficiently high number of strain data sets was available. In addition, a large range (minimum–maximum) of the CV ratio was found for every parameter among the respective strain data sets. This clearly demonstrated the appearance of unpredictable major interactions between genotype and environment regarding the sex-specific variability of the blood parameters analyzed. As no particular parameter is described with a robust difference in the phenotypic variability between both sexes which may be used as a “positive control”, the published increase of the phenotypic variability (= CV) within each sex by 37% in group housed animals [4] may be used to evaluate the results detected in this study. The difference of 37% would result in a CV ratio of 0.39 or 0.61 when comparing single housed mice and group housed mice. This deviation from the hypothesized CV ratio of 0.5 is much higher than the deviations of the mean CV ratios from the hypothesized CV ratio of 0.5 detected in our study for all parameters analyzed where a sufficiently high number of strain data sets was available.

## Limitations

Data sets of the Mouse Phenome Database (https://phenome.jax.org) provide reference values for the inbred strains predominantly used. It is assumed that the projects providing the strain data sets have been carried out by using standardized protocols, but not especially for the analysis of sex-specific phenotypic variability.

## Data Availability

The data sets were downloaded from the Mouse Phenome Database (https://phenome.jax.org) and selected as described in “Methods” section. The accession numbers that refer to the specific datasets used in this study are given as ontology terms (VT, vertebrate trait ontologies) at the beginning of “Methods” section.

## Abbreviations

ALT: Alanine aminotransferase
AP: Alkaline phosphatase
AST: Aspartate aminotransferase
CC: Collaborative Cross
CK: Creatine kinase
CV: Coefficient of variation
MCH: Mean corpuscular hemoglobin
MCHC: Mean corpuscular hemoglobin concentration
MCV: Mean corpuscular volume
RBC: Red blood cell count
VT: Vertebrate trait ontologies
WBC: White blood cell count
